# Antigenic comparison of the neuraminidases from recent influenza A vaccine viruses and 2019–2020 circulating strains

**DOI:** 10.1038/s41541-022-00500-1

**Published:** 2022-07-14

**Authors:** Jin Gao, Xing Li, Laura Klenow, Tahir Malik, Hongquan Wan, Zhiping Ye, Robert Daniels

**Affiliations:** Division of Viral Products, Center for Biologics Evaluation and Research, Food and Drug Administration, Silver Spring, MD 20993 USA

**Keywords:** Inactivated vaccines, Inactivated vaccines, Influenza virus

## Abstract

Although viral-based influenza vaccines contain neuraminidase (NA or N) antigens from the recommended seasonal strains, NA is not extensively evaluated like hemagglutinin (H) during the strain selection process. Here, we compared the antigenicity of NAs from recently recommended H1N1 (2010–2021 seasons) and H3N2 (2015–2021 seasons) vaccine strains and viruses that circulated between September 2019 and December 2020. The antigenicity was evaluated by measuring NA ferret antisera titers that provide 50% inhibition of NA activity in an enzyme-linked lectin assay. Our results show that NAs from circulating H1N1 viruses and vaccine strains for the 2017–2021 seasons are all antigenically similar and distinct from the NA in the H1N1 strain recommended for the 2010–2017 seasons. Changes in N1 antigenicity were attributed to the accumulation of substitutions over time, especially the loss of an *N*-linked glycosylation site (Asn386) in current N1s. The NAs from circulating H3N2 viruses and the 2020–2021 vaccine strains showed similar antigenicity that varied across the N2s in the 2016–2020 vaccine strains and was distinct from the N2 in the 2015–2016 vaccine strain. These data suggest that the recent N1 antigenicity has remained similar since the loss of the head domain *N*-linked glycosylation site, whereas N2 antigenicity has changed more incrementally each season.

## Introduction

The first licensed U.S. influenza vaccine consisted of strains from one influenza A virus (IAV) and one influenza B virus (IBV) that were propagated in chicken eggs, isolated using red blood cells, and inactivated with formalin^1,2^. During the early trials individuals receiving this vaccine were found to generate antibodies capable of inhibiting the receptor binding function of the hemagglutinin (HA or H) antigen and this property was shown to correlate with protection from the vaccine^3^. Around the time hemagglutination-inhibition antibody titres became accepted as a correlate of protection, several studies began showing that antibodies against the less abundant neuraminidase (NA or N) antigen can also limit the severity of influenza infections in animals and humans^4–6^. However, establishing a correlate of protection and quantifying the content in vaccines have proven to be more difficult for NA than HA.

Inactivated viruses are still the most common antigen source for influenza vaccines administered in the U.S^7^. The vaccine viruses are now produced in cells, as well as eggs, and the purification and inactivation processes have improved^8–10^. Most of the current viral-based influenza vaccines are also quadrivalent, containing HA and NA antigens from two recommended IAV and two recommended IBV strains^11^. One of the IAV strain is from the H1N1 subtype and the other is from the H3N2 subtype, whereas the two recommended IBV strains are from the Yamagata and Victoria lineages, respectively. To account for changes in circulating influenza viruses, the recommended vaccine strains are selected each season based on a combination of epidemiological, HA antigenicity and modelling data^12^. Efforts are then made to create high growth reassortant candidate vaccine viruses for cell and egg-based vaccines that possess a HA which is antigenically similar to the HA from the recommended strain.

Despite the continuous strain evaluations and updates, influenza vaccine efficacy remains less than ideal^13,14^, suggesting influenza vaccines may benefit from the inclusion of other protective antigens. Consequently, NA is receiving more consideration as a second antigen for influenza vaccines, especially since NA antibodies can also provide cross protection against strains carrying HAs that are antigenically distinct from the vaccine^15–19^. However, NAs in vaccines are currently not selected, but rather a consequence of the pairing with a recommended HA in an available isolate. As a result, egg and cell-based vaccine strains for the same influenza virus subtype or lineage can contain NAs with different sequences and it is not clear if the changes can impact any potential protection from NA due to the general lack of antigenic data.

Many recent studies have begun to use an immobilised enzyme-linked lectin assay (ELLA) to measure NA inhibition (NAI) antibody titres and to assess NA antigenicity^20–25^. The ELLA was first developed to examine NA activity and inhibition using erythrocyte glycoproteins as a substrate and agglutination by the galactose-binding lectin peanut agglutinin as a reporter^26,27^. This assay has since been modified and the glycans from bovine fetuin are used for measuring viral NA activity in the absence and presence of serum containing NA antibodies^28–32^. An advantage of the ELLA is that it can capture antibody-mediated interference of NA enzymatic activity^33,34^, which has been shown to correlate reasonably well with NA-mediated protection^21,35^. However, in recent years the analysis of H3N2 isolates by the ELLA has been very limited because these strains grow poorly in eggs^36^.

In this study we compared the antigenicity of NA subtypes 1 (N1) and 2 (N2) from recently circulating IAVs and vaccine strains using ferret antisera coupled with ELLA. To eliminate the dependence on field isolates, the analysis was performed using reverse genetic viruses that carry the same HA with NAs from recent H1N1 and H3N2 vaccine strains and circulating strains that were prevalent between September 2019 and December 2020. Our results show that NAs from the circulating strains and the 2020–2021 recommended H1N1 and H3N2 vaccine strains are antigenically similar. They also demonstrated that the antigenicity in NAs from recent H1N1 2009 pandemic-like viruses abruptly changed upon the loss of an *N*-linked glycosylation site in the head domain (Asn386), and that N2 antigenicity appears to be changing more incrementally with each season. The benefit of combining bioinformatics and reverse genetics with the ELLA for evaluating NA antigens in current and future vaccine strains is discussed.

## Results

### Changes in virus NA content do not impact the enzyme-linked lectin assay (ELLA) results

**Fig. 1:**
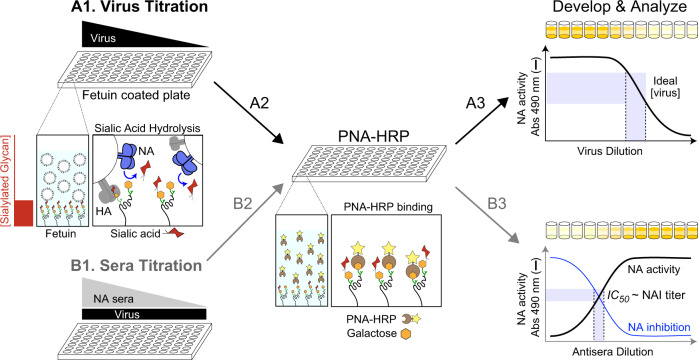
Illustration of the enzyme-linked lectin assay (ELLA) for comparing NA antigenicity. Step A1, viruses are titrated using fetuin-coated plates. Step A2, NA exposed galactose residues on the fetuin glycans are bound by peanut agglutinin linked to horseradish peroxidase (PNA-HRP). Step A3, PNA-HRP binding is determined using the substrate OPD and measuring the absorbance (Abs) at 490 nm. Step B1, Ferret antisera are titrated with a fixed virus dilution from the linear range and added to fetuin-coated plates and processed similarly for PNA-HRP binding (Step B2) and development (Step B3). NA inhibition (NAI) titres are determined by inversely plotting the NA activity data and calculating the reciprocal sera dilution that corresponds to the 50% inhibitory concentration (*IC*_*50*_).

To evaluate NA antigenicity, we combined reverse genetic viruses grown in embryonated chicken eggs with the standard ELLA approach for monitoring the inhibition of NA activity by ferret antisera^31,32,37^. The ELLA approach utilises glycans on immobilised bovine fetuin for the NA substrate, a horseradish peroxidase labelled galactose-binding protein (peanut agglutinin) as a reporter, and viruses for the NA source (Fig. 1). Initially, viruses are titrated in the ELLA to determine a dilution that produces signal in the linear range of the absorbance reading (Fig. 1, steps A1–A3). Ferret serum against a specific NA is then titrated and incubated with the virus at the predetermined concentration prior to performing the ELLA and calculating the reciprocal sera dilution that causes 50% inhibition of the NA activity, which is reported as the NAI titre (Fig. 1, steps B1–B3).

**Fig. 2:**
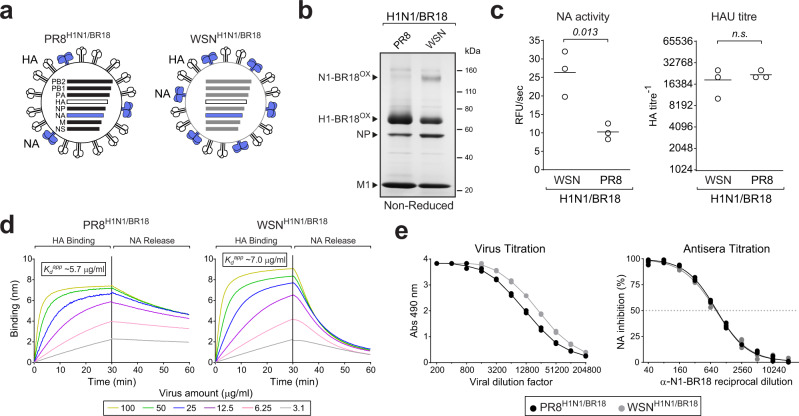
Changes in viral NA content do not alter the ELLA results. **a** Diagram of the PR8^H1N1/BR18^ and WSN^H1N1/BR18^ reassortant viruses. **b** Non-reduced Coomassie stained gel of purified PR8^H1N1/BR18^ and WSN^H1N1/BR18^ viruses (~5 µg). Oxidised (OX) N1-BR18 and H1-BR18 are indicated with the viral proteins NP and M1. Original gel image is provided in Supplementary Fig. 5. **c** NA activities (left panel) and HAU titres (right panel) are shown for three independently purified virus batches with protein concentrations of 1 mg/ml. *P* values from an unpaired student *T* test, 95% CI, are displayed. *n.s*.- not significant. **d** Binding of purified PR8^H1N1/BR18^ (left panel) and WSN^H1N1/BR18^ (right panel) virions to fetuin was measured by bio-layer interferometry. Curves were generated with equal densities of immobilised fetuin and the indicated purified virus protein concentrations. HA binding was measured in the presence of 10 µM zanamivir to inhibit NA activity and calculate the apparent dissociation constant (*K*_*d*_^*app*^) for fetuin based on total viral protein concentrations. To follow NA-mediated release, biosensors with bound viruses were transferred to a well without zanamivir. **e** ELLA virus titrations for the PR8^H1N1/BR18^ and WSN^H1N1/BR18^ viruses (left panel) are displayed with the ferret α-N1-BR18 antisera titration data (right panel) used to calculate the NAI titre. Virus titration data is from a single analysis run in duplicate. Antisera titration data is from two independent runs performed in duplicate. Source data for (**c**–**e**) are provided in the source data file.

Although generating reverse genetic viruses eliminates the dependence on strain isolates and minimises the potential interference by antibodies against the inoculating strain HA^38,39^, NA content in the viruses are still likely to vary. Therefore, we initially compared the ELLA results from two purified reassortant viruses (PR8^H1N1/BR18^ and WSN^H1N1/BR18^) that possess different amounts of HA and NA antigens from the H1N1 strain A/Brisbane/02/2018 (Fig. 2a)^25^. Based on Coomassie stained gels (Fig. 2b), NA activities and hemagglutination unit (HAU) titres (Fig. 2c), the WSN^H1N1/BR18^ virus was estimated to contain ~2.5 times more NA and slightly less HA than the PR8^H1N1/BR18^ virus. Supporting these properties, NA-mediated release of WSN^H1N1/BR18^ from fetuin was faster than PR8^H1N1/BR18^, whereas the HA binding affinity was slightly less (Fig. 2d). The ELLA captured the ~2-fold higher NA content in the WSN^H1N1/BR18^ virus during the virus titration (Fig. 2e, left panel) and ferret antisera against N1-BR18 produced similar NAI titres (~800) for both viruses (Fig. 2e, right panel, and Table 1), confirming our experimental set-up accounts for changes in the viral NA content when the HAs are identical.

**Table 1:** NAI titres from the indicated reassortant viruses and antisera.

	*α-N1-BR18*	*α-N1-GD19*	*α-N2-HK19e*	*α-N2-HK19c*
WSN^H1N1/BR18^	**833**	n.d.	n.d.	n.d.
PR8^H1N1/BR18^	**812**	n.d.	n.d.	n.d.
PR8^N1/GD19^	n.d.	**4443**	<10	14
PR8^N2/HK19e^	n.d.	<10	**140**	133
PR8^N2/HK19c^	n.d.	<10	85	**310**

**Fig. 3:**
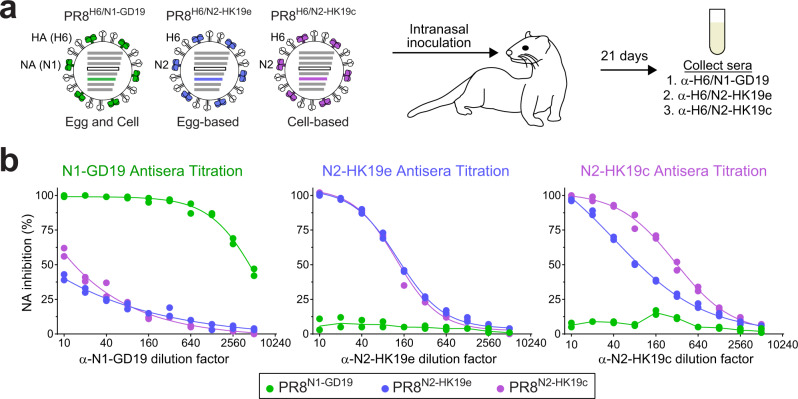
Characterisation of ferret antisera against the NAs in the 2020–2021 IAV vaccine strains. **a** Diagram for generating ferret antisera against the NA antigens (N1-GD19, N2-HK19e, and N2-HK19c) from the recommended egg and cell-based IAV vaccine strains for the 2020–21 northern hemisphere season. Ferret sera were collected 21 days post intranasal inoculation with PR8 double gene reassortant viruses carrying H6 and one of the vaccine strain NAs. **b** Specificity and reactivity of each ferret antiserum was assessed by the ELLA using PR8 single gene reassortant viruses that carry the IAV vaccine strain NAs. Representative data from a single analysis performed in duplicate are displayed. Source data are provided in the source data file.

NAI titres represent the mean *IC*_*50*_ for each ferret antiserum (italics) against the indicated viruses that were determined from an ELLA performed in duplicate. Titres from reassortant viruses carrying a NA that matches the sequence of the NA used to generate the ferret antiserum are in bold. NAI titres that could not be measured are listed as less than the lowest reciprocal serum dilution. Source data are provided in the source data file (Figs. 2e and 3b).

*n.d.* not done.

### Characterisation of ferret antisera against N1 and N2 from the 2020–2021 vaccine strains

For the 2020–2021 northern hemisphere influenza season, the recommended H1N1 strains for egg-based (A/Guangdong-Maonan/SWL1536/2019) and cell-based (A/Hawaii/70/2019) vaccines contain NAs with identical amino acid sequences (Supplementary Fig. 1). However, the recommended H3N2 strains for egg-based (A/Hong Kong/2671/2019) and cell-based (A/Hong Kong/45/2019) vaccines possess NAs that differ at positions 45 and 469 (Supplementary Fig. 2). Therefore, we generated ferret antiserum against one N1 vaccine antigen (N1-GD19) and the N2 antigens from the egg (N2-HK19e) and cell-based (N2-HK19c) vaccine strains. To minimise the potential for poor NA responses due to the low abundance in virions and the labile nature of NA^40^, each antiserum was produced by intranasal inoculation with a PR8 double gene reassortant virus that carries a HA subtype 6 (H6) and one of the NA vaccine antigens (Fig. 3a).

Specificity of the antisera was confirmed using PR8 single gene reassortant viruses containing the NA vaccine antigens. The N1-GD19 antiserum showed high reactivity with PR8^N1-GD19^, producing an NAI titre of ~4400, and minimal reactivity with viruses carrying the N2 vaccine antigens (Fig. 3b and Table 1). Antisera against N2-HK19e and N2-HK19c produced modest NAI titres of 140 and 310 for their respective matching strains and did not react with PR8^N1-GD19^ (Fig. 3b and Table 1). Interestingly, both N2 antiserum showed a bias for the matching N2 antigen (Table 1), suggesting residues 45 or 469 could be in an epitope recognised by NAI antibodies.

### Comparison of NAs from recent H1N1 viruses and the 2020–2021 H1N1 vaccine strain

**Fig. 4:**
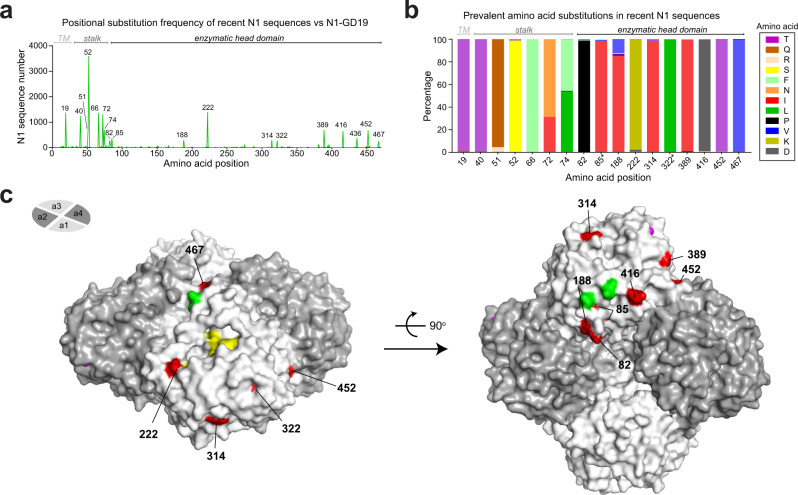
Comparison of NAs from recent H1N1 viruses and the 2020–2021 vaccine strain. **a** Amino acid positional analysis displaying the number of recent N1 sequences that encode different amino acids from N1-GD19 at each position. N1 sequences were from human H1N1 IAVs collected between September 1, 2019 and December 15, 2020. Regions corresponding to the transmembrane (TM) domain, stalk and enzymatic head domain are indicated. **b** Bar graph displaying the prevalence of the amino acids in the recent N1 sequences that differ from N1-GD19 at each position. Only amino acids visible in the graph are listed in the legend. Asterisk indicates amino acids with little surface exposure. **c** Amino acid positions that differ in the recent N1 sequences are highlighted (red) on a single monomer of a head domain tetramer (PDB ID: 3NSS)^54^ along with the Asn residues (green) of the conserved N-linked glycosylation sites^48^, NA active site residues (yellow), and Ca^2+^ ions (pink). Side (left) and bottom (right) views of the NA tetramer were generated using Pymol.

To identify potential antigenic changes between NAs from circulating H1N1 IAVs and the 2020–2021 vaccine strain, NA sequences collected between September 1, 2019 and December 15, 2020 were compared to N1-GD19. A positional analysis showed that amino acid substitutions in the recent N1 sequences are more common in the stalk region than the enzymatic head domain (Fig. 4a), and that the substitutions generally involved a change to a specific amino acid (Fig. 4b). Mapping the prevalent substitution sites on a structure of the N1 head domain revealed that most are surface exposed. One of the sites (467) is positioned on the top of the tetramer, another (222) is near the active site, and several are located on the side and bottom of the tetramer where the head is linked to the stalk (Fig. 4c). The surface exposure and the proximity of at least one substitution to the NA active site, indicated that these common N1 substitutions could result in an antigenic change from N1-GD19.

Protective epitopes in NA have been almost exclusively mapped to the head domain^22,23,33,41,42^. Therefore, we used this region to identify several recent N1s that are good candidates for an antigenic comparison to N1-GD19. Approximately 66% of N1 head domain sequences were either identical to N1-GD19, or possessed substitutions at 1, 2, 5, or 6 specific positions in the head domain (Supplementary Fig. 3a). For each of these substitution groups we identified N1s that contained the most common amino acid position changes (Supplementary Fig. 3b), generated PR8 reassortant viruses, and examined the reactivity to ferret antisera against N1-GD19. The NAI titres for the N1-GD19 ferret antiserum indicated that the four representative N1s are antigenically similar to N1-GD19 as only the N1 with six head domain substitutions (N1-NJ20) showed a lower (twofold reduction) in NAI titre (Table 2), suggesting the NA antigen in the H1N1 vaccine strain for the 2020–2021 season is a reasonable choice.

**Table 2:** NAI titres for the indicated N1 reassortant viruses and antisera.

	*α-N1-GD19*	*α-N1-BR18*	*α-N1-MI15*	*α-N1-CA09*
PR8^N1-AZ20^	3493	960	498	143
PR8^N1-Ont20^	3082	720	518	121
PR8^N1-NC20^	3063	873	470	114
PR8^N1-NJ20^	1809	538	271	95
PR8^N1-GD19^	**3885**	1126	582	151
PR8^N1-BR18^	4762	**1043**	536	142
PR8^N1-MI15^	5379	1454	**787**	194
PR8^N1-CA09^	246	110	71	**798**

NAI titres represent the mean *IC*_*50*_ values for each ferret antiserum (italics) against the indicated viruses that were determined from an ELLA performed in duplicate. Titres from PR8 reassortant viruses where the N1 sequence matches the N1 used to produce the ferret antisera are in bold. All ferret antisera were produced via intranasal inoculation with PR8 reassortant viruses carrying H6 with the indicated NA. Source data are provided in the source data file.

### Loss of an N-linked glycan site altered the antigenicity of NAs in recent H1N1 vaccine viruses

**Fig. 5:**
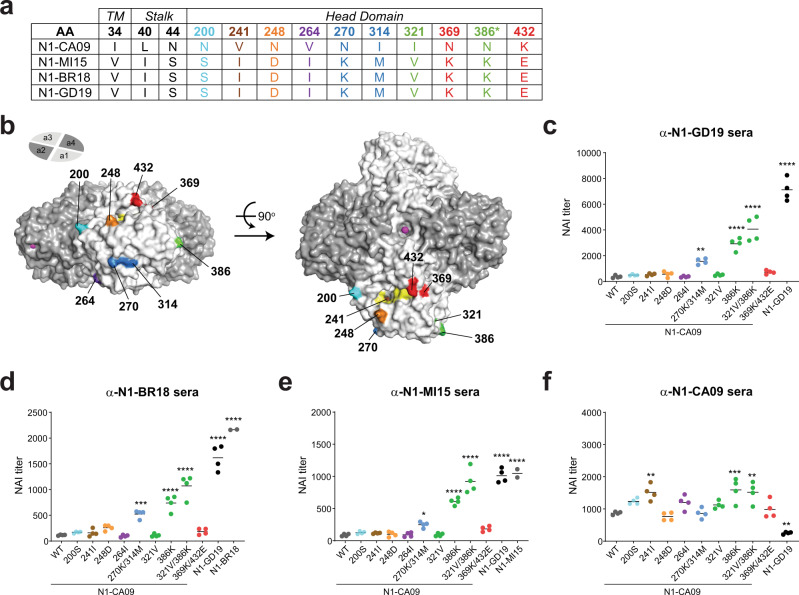
Head substitutions contribute to the antigenic change in NAs from recent H1N1 vaccine strains. **a** Chart displaying the amino acid positions where N1-GD19, N1-BR18 and N1-MI15 all differ from N1-CA09 by the same amino acid. Positions that were not analysed experimentally are shown in black. The asterisk indicates the position where N1-CA09 has an N-linked glycosylation site. **b** N1 head domain structure (PDB ID: 3NSS)^54^ showing the positions of the residues that are unique in N1-CA09 on a single NA monomer. Active site residues (yellow) and Ca^2+^ ions (pink) are also depicted. Side (left) and top (right) views of the NA tetramer were generated using Pymol. **c**–**f** Graphs displaying the NAI titers for the indicated NAs that were determined using ferret sera against (**c**) N1-GD19, (**d**) N1-BR18, (**e**) N1-MI15, and (**f**) N1-CA09. NAI measurements were made in duplicate in two independent runs using PR8 single gene reassortant viruses carrying the indicated NAs. N1-BR18 and N1-MI15 controls were run once in duplicate. *P* values were determined using a one-way ANOVA Dunnett’s multiple comparison test with a 95% CI and N1-CA09 as the comparator. **P ≤ 0.01; ***P ≤ 0.001; ****P ≤ 0.0001. Source data for (**c**–**f**) are provided in the source data file.

When we extended the antigenic comparison to NAs from the three preceding H1N1 vaccine viruses a distinct pattern emerged. All recent N1s displayed similar antigenicity to the NAs (N1-BR18 and N1-MI15) from the recommended H1N1 vaccine strains for the 2017–2020 seasons and very little similarity to the NA (N1-CA09) in the recommended H1N1 vaccine strain for the 2010–2017 seasons (Table 2). To determine if a specific substitution is responsible for the antigenic change, we compared the NA sequences from the four recent H1N1 vaccine strains (Supplementary Fig. 1) and focused on substitutions in the head domain that are unique for N1-CA09. Ten sites were found and one corresponded to an *N*-linked glycosylation site in N1-CA09 (N386) that was lost in N1-MI15, N1-BR18 and N1-GD19 (Fig. 5a), as it became less prevalent in strains isolated after 2013^23^. Four of the sites mapped to distinct areas in the N1 head domain and the remaining six, including the glycosylation site substitution, clustered in three separate pairs (Fig. 5b).

The identified substitutions were introduced into N1-CA09 individually, or in pairs, and PR8 single gene reassortant viruses were generated. Viruses carrying the 270 K/314 M, 386 K and 321 V/386 K substitutions all showed higher NAI titres with sera against N1-GD19, N1-BR18 and N1-MI15 (Fig. 5c–e), suggesting these substitutions contributed independently or additively to the antigenic change in the recent N1s. The largest single substitution antigenic change was from 386 K, which results in the loss of an *N*-linked glycosylation site in N1-CA09. The change was more pronounced with the addition of the 321 V substitution that is located beneath residue 386 in the structure, confirming that the potential glycosylation of N386 masks a viable NAI epitope that is recognised in ferrets. Despite the observed changes, none of the N1-CA09 variants showed the same reactivity as N1-GD19 with the sera against N1-GD19 or N1-BR18, indicating the antigenic difference between N1-CA09 and the recent N1s is due to a combination of the lost glycosylation site and other substitutions. Interestingly, none of the substitutions in N1-CA09 caused a loss of reactivity with serum against N1-CA09, suggesting the low reactivity of N1-GD19 is either due to multiple substitutions or the few substitutions we did not examine.

### NAs from recent H3N2 viruses and vaccine strains show more antigenic variability

**Fig. 6:**
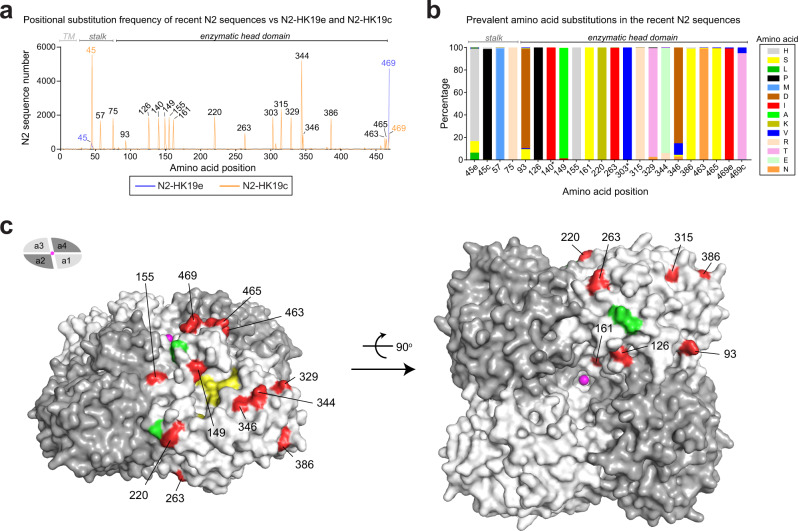
Comparison of NAs from recent H3N2 viruses and the 2020–2021 vaccine strains. **a** Amino acid positional analysis displaying the number of recent N2 sequences that encode amino acids which differ from N2-HK19c (orange) and N2-HK19e (blue) at each position. N2 sequences were from human H3N2 IAVs isolated between September 1, 2019 and December 15, 2020. Amino acid positions corresponding to the TM domain, stalk and enzymatic head are indicated. **b** Bar graph displaying the prevalence of the amino acids in recent N2 sequences that differ from N2-HK19e and N2-HK19c at each position. Note N2-HK19e and N2-HK19c differ at positions 45 and 469. Only amino acids visible in the graph are listed. Asterisk indicates amino acids with little surface exposure. **c** Amino acid positions that differ in recent N2 sequences were highlighted (red) on a single monomer of a head domain tetramer (PDB ID: 4GZT)^55^. Asn residues (green) of the conserved N-linked glycosylation sites^48^, NA active site residues (yellow), and Ca^2+^ ions (pink) are also depicted. Positions 140 and 303 are not visible. Side (left) and bottom (right) views of the NA tetramer were generated using Pymol.

We performed a similar analysis to determine if any potential antigenic changes exist between the NA sequences from recent H3N2 IAVs and the 2020–2021 vaccine strains. Due to the sequence differences, N2-HK19e and N2-HK19c were both used as references for the positional analysis. In contrast to N1, amino acid changes in recent N2s were common in the head domain (Fig. 6a). We also observed that the C-terminal residue of N2-HK19e (469 T) and the stalk residue of N2-HK19c (45 S) are not prevalent and that the amino acid differences in the recent N2s generally involved a change to a specific amino acid (Fig. 6b). Mapping on a structure of the N2 head domain showed that most substitutions correspond to surface exposed residues and that many of them are positioned around the active site and on the top of the tetramer near the central Ca^2+^ binding site (Fig. 6c), which has been shown to be critical for NA activity^40^.

To identify recent N2 sequences for the antigenic comparison to N2-HK19e and N2-HK19c we again focused on the head domain and found that very few sequences were identical to either N2-HK19e or N2-HK19c. Tabulating the most prevalent substitution combinations in the head domain (with respect to N2-HK19c) revealed that ~55% of the recent N2 sequences contain amino acid substitutions at 2, 4, 5, 6, or 10 specific positions in the head domain (Supplementary Fig. 4a). From this analysis eight N2s with the most common head domain substitution combinations were identified (Supplementary Fig. 4b). We then generated PR8 single gene reassortant viruses for all eight NAs and tested the reactivity to ferret antisera against N2-HK19e, N2-HK19c, and the NAs from the four preceding H3N2 vaccine strains.

With the sera against N2-HK19e and N2-HK19c no significant decrease was observed between the NAI titres of the recent N2s and the vaccine strain matched N2s, indicating they all possess reasonable antigenic similarity (Table 3). Interestingly, all recent N2s that possessed the 469 T substitution showed higher reactivity with the sera against N2-HK19e than N2-HK19c, indicating the C-terminus is part of an antigenic domain for NAI antibodies in N2. For the serum against N2-KS17, only the N2 with 10 substitutions (N2-Que20) showed similar antigenicity, which was not surprising as the sequence is almost identical to N2-KS17 and they both displayed similar NAI titres with all sera that were often very high. The sera against N2-SG16 and N2-HK14 showed more variability as the NAI titres for some recent N2s were close to those for the matching vaccine strain N2s. For the serum against N2-SW13, the recent N2s displayed very little reactivity, indicating they are all antigenically distinct from the NA in the H3N2 vaccine strain for the 2015–2016 season. Together, these results imply that the NAs in the recent H3N2 viruses matched reasonably well to those in the 2020–2021 vaccine strains and that the antigenicity of recent N2s is more variable and less defined than the antigenicity of the recent N1s.

**Table 3:** NAI titres from the indicated N2 reassortant viruses and antisera.

	*ɑ-N2-HK19c*	*ɑ-N2-HK19e*	*ɑ-N2-KS17*	*ɑ-N2-SG16*	*ɑ-N2-HK14*	*ɑ-N2-SW13*
PR8^N2-VA20 a^	124	143	29	77	62	21
PR8^N2-MN20^	270	152	99	117	87	22
PR8^N2-MI20^	231	129	53	44	37	8
PR8^N2-PA20 a^	103	149	27	31	24	8
PR8^N2-CA20^	420	267	139	175	151	34
PR8^N2-OH20 a^	167	178	47	100	101	17
PR8^N2-MA20 b^	292	111	77	138	110	22
PR8^N2-Que20 c^	2509	668	605	725	765	98
PR8^N2-HK19c^	**333**	189	96	123	86	25
PR8^N2-HK19e^	160	**213**	37	95	83	22
PR8^N2-KS17^	2181	909	**729**	713	598	99
PR8^N2-SG16^	199	99	75	**104**	64	27
PR8^N2-HK14^	19	68	9	24	**239**	245
PR8^N2-SW13^	30	72	12	32	342	**336**

NAI titres represent the mean *IC*_*50*_ values for each ferret antiserum (italics) against the indicated viruses that were determined from an ELLA performed in duplicate. Titres from PR8 reassortant viruses where the N2 sequence matches the N2 used to generate the ferret antisera are in bold. All ferret antisera were produced by intranasal inoculation with PR8 reassortant viruses carrying H6 with the indicated NA. Source data are provided in the source data file.

^a^N2 possesses the 469 T substitution present in N2-HK19e.

^b^N2 contains a potential N-linked glycosylation site at 463 N.

^c^N2 sequence is more similar to N2-KS17 than N2-HK19c or N2-HK19e.

## Discussion

During previous influenza seasons our lab has supported the influenza A vaccine strain selection process by using available isolates to evaluate circulating NA antigens. The isolate dependence severely impaired these evaluations because only a few H1N1 and almost no H3N2 strains were received, partly due to the poor growth of recent H3N2 strains in eggs. In addition, the few isolates we did obtain often arrived close to the February strain selection meeting and many carried NAs with identical sequences. To perform a more systematic evaluation of the NAs in circulating strains, we changed our strategy for the 2020–2021 season to a sequence-based approach that used reverse genetic viruses for the NA source. The results showed that the NA antigens in the recommended H1N1 and H3N2 strains for egg and cell-based vaccines for the 2020–2021 season are antigenically similar to the majority of NAs in the sequenced circulating H1N1 and H3N2 viruses. They also indicated that the loss of N-linked glycans and the C-terminus of N2 can potentially influence NA antigenicity. In contrast to prior seasons, these conclusions were based on data that covered ~66% of the H1N1 strains and ~55% of the H3N2 strains that were collected between September 2019 and December 2020. As this platform is expanded, we envisage that it will aid in the selection of NA antigens for recombinant protein- and mRNA-based vaccines, and in the selection between vaccine strains that carry similar HAs but different NAs.

By including sera against NAs from prior vaccine strains we were able to observe a clear difference in the antigenicity of NAs from recent H1N1 strains as they reacted well with all sera except for that against N1-CA09. One of the main contributors to the antigenic change was the loss of an *N*-linked glycosylation site (Asn386) that was present on N1-CA09, supporting a prior study that inferred this site can influence N1 antigenicity based on a comparison of NAs from H1N1 strains isolated between 2009 and 2016^23^. We showed that the reactivity further enhanced when it was combined with a second substitution (321) that has little surface exposure and is largely located under residue 386 in the structure. Another antigenic site included residues 270 and 314, which are on the opposing face of the monomer in a region that has been shown to be recognised by both mouse and human NAI antibodies^33,43^. While this suggests these two sites (386 and 270/314) could be independent NAI epitopes, it does not rule out the possibility that the 270/314 substitutions have an additive effect on a distal epitope that is masked by the glycan at Asn386. Glycan masking has been reported for numerous antigens including NA^44–47^, making it interesting that the N1-CA09 serum failed to recognise the more recent N1s lacking the *N*-linked glycosylation site. The N1-CA09 serum reactivity was also not impaired by any of the N1 substitutions found in the later vaccine strains, suggesting that the NAI epitopes in N1-CA09 require multiple substitutions to be disrupted.

More antigenic variability was observed in the NAs from the recent H3N2 strains as every serum reacted with at least one N2 at a level that was similar to the N2 used to generate the sera. There also was no clear temporal distinction as some recent N2s showed more reactivity with sera against N2-HK14 and N2-SG16 than N2-KS17. The data for the N2s from the egg and cell-based vaccine strains also revealed that the C-terminus of N2 can influence an antigenic domain for NAI antibodies. This interpretation, which suggests the cell-based N2 with 469 I is a slightly better antigenic match for circulating H3N2 strains, was based on the reproducible bias of the two antisera for N2s with an identical C-terminus and previous work demonstrating the amino acid at position 468 can influence NAI titres for N2^44^. Interestingly, the N2 C-terminus is near the central Ca^2+^-binding site that was previously shown to be crucial for N1 enzymatic activity^40^, raising the possibility that antibody binding decreases the Ca^2+^ affinity of this site, thereby reducing N2 activity. Although more directed studies are needed to determine the precise mechanism of inhibition, these observations indicate the N2 C-terminus should receive some consideration during H3N2 strain selection. This especially applies to the 2021–22 season where the recommended H3N2 vaccine strains carry a NA with a C-terminal N-linked glycosylation site at position 463, which is distinct from the reported variable glycosylation sites in N2^48^ and had little data demonstrating its prevalence at the time of strain selection.

There are still some significant deficiencies in the ELLA-based antigenic characterisation of NA. The most striking are the false positive NAI titres that are obtained from antibodies against HA^38,39^, the time consuming nature of the assay, and the significantly lower NAI titres that are obtained for N2 compared to N1, resulting in a smaller dynamic range for evaluating N2. Despite these deficiencies, systematic approaches are likely the most effective way for evaluating antigenic drift in NA and the current ELLA has been shown to correlate reasonably well with protection in humans^21,35^. However, it is still unclear how well naïve ferrets model NA epitope recognition by humans or how valuable sera from vaccinated individuals is for assessing NA responses due to the limited NA content in approved vaccines and the potential influence of the manufacturing process on the antigenicity of this labile protein. Future studies and modifications to address these deficiencies will improve this approach for evaluating NA antigenicity and increase the likelihood that NAs in circulating viruses are evaluated as thoroughly as the HA antigens during the vaccine selection process.

## Methods

### Reagents

Madin-Darby canine kidney 2 cells (MDCK.2; CRL-2936) and HEK 293 T/17 cells (CRL-11268) were purchased from LGC Standards. Dulbecco’s Modified Eagles Medium (DMEM), L-glutamine, penicillin/streptomycin (P/S), Opti-MEM I (OMEM), Simple Blue Stain, Novex 4–12% Tris-Glycine SDS-PAGE gels, Maxisorp 96-well plates and Lipofectamine 2000 transfection reagent were obtained from Thermo Fisher Scientific. Fetal bovine serum (FBS), 2′-(4-methylumbelliferyl)-α-d-*N*-acetylneuraminic acid (MUNANA) and Zanamivir were acquired from Atlanta Biologicals, Cayman Chemical and Moravek Inc, respectively. Bovine fetuin, *o*-phenylenediamine dihydrochloride (OPD), and HRP-linked peanut agglutinin were purchased from Sigma. Specific-pathogen-free (SPF) eggs and turkey red blood cells (TRBCs) were purchased from Charles River Labs and the Poultry Diagnostic and Research Center (Athens, GA), respectively.

### Plasmids and constructs

Reverse genetics (RG) plasmids containing the PR8 gene segments, provided by Dr. Robert Webster (St. Jude Children’s Research Hospital), were sequenced prior to use and correspond to the following GenBank Identifications: CY038902.1 (PR8-PB2), CY038901.1 (PR8-PB1), CY084019.1 (PR8-PA), CY146825.1 (PR8-HA), CY038898.1 (PR8-NP), MH085246.1 (PR8-M), and CY038899.1 (PR8-NS). RG plasmids containing the NA and HA genes from the following influenza strains were created in previous studies: A/H1N1/California/07/2009 (N1-CA09), A/H1N1/Michigan/45/2015 (N1-MI15), A/H1N1/Brisbane/02/2018 (N1-BR18) and A/H6N2/turkey/Massachusetts/3740/1965 (H6)^40,49,50^. NA genes from the following influenza strains were synthesised and inserted into the pHW2000 plasmid^51^ by Genscript: A/H1N1/Guangdong-Maonan/SWL1536/2019 (N1-GD19), A/H1N1/Ontario/RV4818/2020 (N1-Ont), A/H1N1/Arizona/12100/2020 (N1-AZ20), A/H1N1/North Carolina/RV4818/2020 (N1-NC20), A/H1N1/New Jersey/11588/2020 (N1-NJ20), A/H3N2/Hong Kong/45/2019 (N2-HK19c), A/H3N2/Hong Kong/2671/2019 (N2-HK19e), A/H3N2/Virginia/12037/2020 (N2-VA20), A/H3N2/Minnesota/24/2020 (N2-MN20), A/H3N2/Michigan/157/2020 (N2-MI20), A/H3N2/Pennsylvania/03/2020 (N2-PA20), A/H3N2/California/33/2020 (N2-CA20), A/H3N2/Ohio/12141/2020 (N2-OH20), A/H3N2/Massachusetts/07/2020 (N2-MA20), and A/H3N2/Quebec/RV3597/2020 (N2-Que20). RG plasmids encoding N1-CA09 with single and double point mutations were generated by site directed mutagenesis (Agilent). All RG plasmids were confirmed by sequencing before use (FDA core facility).

### Cell culture, reverse genetics and viral propagation

MDCK.2 and HEK 293 T/17 cells were maintained at 37 °C with 5% CO_2_ and ~95% humidity in DMEM containing 10% FBS and 100 U/ml P/S. Reassortant viruses were created by 8-plasmid RG^51^ in 6-well plates using the indicated NA, or NA and HA pair, and the complimentary seven, or six, PR8 gene segments. The day before each well received ~2 × 10^6^ 293 T and ~1 × 10^6^ MDCK.2 cells. Eight plasmids (1 µg of each) were added to 200 μl of OMEM, mixed with 18 µl of lipofectamine, and incubated 45 min at room temperature. Wells were washed with 2 ml OMEM, mixtures were added to each well and incubated 5 min at 37 °C prior to adding 800 μl of OMEM. At 24 h post-transfection 1 ml OMEM containing 4 µg/ml TPCK trypsin was added to each well. Culture medium containing the rescued viruses was harvested ~96 h post-transfection and clarified by sedimentation (2000 × g; 5 min) prior to being used to inoculate 10-day old embryonic SPF chicken eggs (100 μl per egg). Eggs were incubated for 3 days at 33 °C, placed at 4 °C for 2 h, the allantoic fluid was collected, clarified by centrifugation (2000 × *g*; 5 min), aliquoted and stored at −80 °C. For purification, viruses in allantoic fluid were inactivated with 0.05% BPL for ~16 h at 4 °C. Prior to use each viral stock was confirmed using MiSeq RNA deep sequencing (FDA core facility).

### Viral purification and SDS-PAGE

BPL inactivated PR8^H1N1-BR18^ and WSN^H1N1-BR18^ reassortant viruses were purified from allantoic fluid by ultracentrifugation^25^. Viruses were isolated by sedimentation (100,000 × g; 45 min) at 4 °C through a 5 ml sucrose cushion (25% w/v sucrose, PBS pH 7.2 and 1 mM CaCl_2_). Pelleted viruses were resuspended in 500 µl sucrose 12.5% w/v in PBS pH 7.2 containing 1 mM CaCl_2_, layered on top of a discontinuous sucrose gradient with four 8.5 ml sucrose layers (60% w/v, 45% w/v, 30% w/v and 15% w/v in PBS pH 7.2 and 1 mM CaCl_2_) and centrifuged at 100,000 × *g* for 2 h at 4 °C. Fractions with a density between 30 and 50% sucrose w/v were pooled, mixed with 2 volumes of PBS pH 7.2 and 1 mM CaCl_2_, and centrifuged (100,000 × *g*; 45 min). The supernatant was discarded, sedimented viruses were resuspended in PBS pH 7.2 containing 1 mM CaCl_2_, protein concentration was determined using a BCA protein assay kit (Pierce) and adjusted to a concentration of 1 mg/ml using PBS pH 7.2 containing 1 mM CaCl_2_, aliquoted and stored at −80 °C. For SDS-PAGE, purified viruses (~5 µg total viral protein) were mixed with 2× sample buffer, heated at 50 °C for 5 min and resolved on a 4–12% polyacrylamide Tris-Glycine SDS-PAGE wedge gel. Gels were stained with simple blue and imaged with an Azure C600 Bioanalytical Imaging System (Azure Biosystems).

### Bio-layer interferometry

Biotinylated fetuin was made using the EZ-Link NHS-PEG4-Biotinylation kit (Thermo Scientific). Briefly, bovine fetuin (Sigma) was dissolved in PBS pH 7.2 to a concentration of 10 mg/ml and incubated with 2.9 mM of the biotinylation reagent at room temperature for 60 min. Biotinylated fetuin was then isolated using an Agilent 1260 Infinity II HPLC equipped with a 21.2 × 300 mm AdvanceBio SEC 300 Å column, a variable wavelength detector and PBS 7.2 as the mobile phase. Fractions containing fetuin were pooled, the protein concentration was determined using BCA, and the labelling was calculated to be ~8 biotin/molecule of fetuin. Binding of the purified BPL inactivated PR8^H1N1-BR18^ and WSN^H1N1-BR18^ viruses to immobilised biotinylated fetuin was performed using a ForteBio Octet Red96 bio-layer interferometer equipped with ForteBio High Precision Streptavidin biosensors at 25 °C with a plate shaking speed of 1000 rpm. Baselines were established for 2 min in 200 μl of buffer (PBS pH 7.2 containing 1 mM CaCl_2_ and 0.005% Tween20) prior to and post incubation of biosensors with 200 μl of 10 nM biotinylated fetuin for 10 min. Fetuin loaded biosensors were then transferred to wells containing 200 μl of the indicated concentrations of the WSN^H1N1-BR18^ or PR8^H1N1-BR18^ viruses in buffer with 10 μM zanamivir for 30 min to analyse HA-mediated virus association. Biosensors were then transferred to new wells containing 200 μl of buffer for 20 min to examine NA-mediate dissociation.

### NA activity and HAU measurements

NA activity measurements were performed using equal amounts of total viral protein in 96-well low protein binding black clear bottom plates (Corning)^25^. Each virus was mixed with 37 °C reaction buffer (0.1 M KH_2_PO_4_ pH 6.0 and 1 mM CaCl_2_) to a volume of 195 μl. Reactions were then initiated by adding 5 μl of 2 mM MUNANA and the fluorescence was measured on a Cytation 5 (Biotek) plate reader at 37 °C for 10 min using 30 s intervals with 365 nm excitation and 450 nm emission wavelengths. Activities were determined by calculating the slope of the early linear region of the emission versus time graph. HAU titres were determined by twofold serial dilutions in 96-well plates with a sample volume of 50 µl. Following dilution in PBS pH 7.2, 50 µl of 0.5% TRBCs were added to each well and incubated 30 min at room temperature. HAU titres corresponded to the last well where agglutination was observed.

### Ferret inoculation and serum collection

All animal experiments were approved by the U.S. FDA Institutional Animal Care and Use Committee under Protocol #2001-05. The animal care and use protocol meets National Institutes of Health guidelines. Intranasal inoculations were performed on 14 week-old male ferrets using allantoic fluid that contained the indicated reassortant virus at an infectious titre between 10^6^ and 10^8^ TCID50/ml on MDCK cells. The inoculum (~0.5 ml) was equally dispensed between both nostrils of the anaesthetised ferret using a 1 ml syringe equipped with a 20 Gauge feeding needle. Ferret temperatures, monitored using an implanted microchip, and body weight were recorded daily for 2 weeks. The sera was harvested 3 weeks post-infection, aliquoted and stored at −80 °C.

### ELLA and NAI titre determination

The ELLA was performed in two parts on 96-well Maxisorp plates coated with 2.5 µg/well of bovine fetuin^31^. Briefly, correct virus concentrations for the ELLA were first determined using twofold serial dilutions of each virus. The dilution series was added to fetuin-coated plates and incubated at 37 °C overnight. Plates were washed and incubated with ~100 ng/well of horse radish peroxidase-linked peanut agglutinin (PNA-HRP) for 2 h at room temperature. Plates were washed again and developed with OPD (0.5 ng/well) for 10 min, the reaction was stopped with 100 µl/well 1 N sulfuric acid and the absorbance at 490 nm was measured. Absorbance was plotted versus the virus dilution and the dilution corresponding to the top of the linear part of the titration curve was chosen. Next, a twofold dilution series was made for each antiserum and transferred to a fetuin-coated plate prior to adding the virus at the concentration determined in the first assay. Plates were incubated at 37 °C overnight, washed, incubated with PNA-HRP and developed as described above. The absorbance for each antiserum and virus pair was background corrected using the average signal obtained from wells containing no virus or serum. To calculate the NAI percentage each background corrected sample signal was subtracted from the average virus control well signal (wells that received virus but no antiserum) and the difference was divided by the average virus control well signal and multiplied by 100%. The NAI percentage was then plotted versus the reciprocal dilution of the antiserum in GraphPad Prism 8.0 and the IC_50_ value, which corresponds to the NAI titre, was determined by a four-parameter least squares fit analysis.

### Sequence analysis

The available NA protein sequences collected from human H1N1 (*N* = 6844) and H3N2 (*N* = 5566) viruses since September 1, 2019 were downloaded from the Global Initiative on Sharing All Influenza Data (GISAID) database on December 15, 2020. Sequences longer or shorter than 469 amino acids were excluded along with those that contained ambiguous amino acid identities. All analyses were performed based on positions in the N1 or N2 amino acid sequences using the indicated vaccine NA sequence as a reference. Results specific for the head domain were obtained by examining the residues corresponding to amino acids 82–469, excluding the stalk and transmembrane domain^52,53^.

### Statistical analysis

Statistical analysis was performed with GraphPad Prism 8 software with the assumptions that the data follows a Gaussian distribution. Student’s unpaired *t*-test was performed using a two-tailed analysis and a confidence interval (CI) of 95%. One-way ANOVA was performed using Dunnett’s multiple comparisons test with a CI of 95%.

### Reporting summary

Further information on research design is available in the Nature Research Reporting Summary linked to this article.

## Supplementary information

Supplementary Figures

REPORTING SUMMARY

## Data Availability

The source data underlying Figs. 2c–e, 3b, 5c–f and Tables 1–3 are provided as a Source Data file. Note the Table 1 source data was determined using the source data for Figs. 2e and 3b. The original image of the gel shown in Fig. 2b is provided in Supplementary Fig. 5.

## Source data

Source Data file
