# Supplementary material for: Antigenic comparison of the neuraminidases from recent influenza A vaccine viruses and 2019–2020 circulating strains
Source: NPJ Vaccines. 2022 Jul 14;7:79. doi: 10.1038/s41541-022-00500-1 (PMC9283437; doi:10.1038/s41541-022-00500-1)
Supplement: Supplementary file 1 — Supplementary Figures [file 41541_2022_500_MOESM1_ESM.pdf]

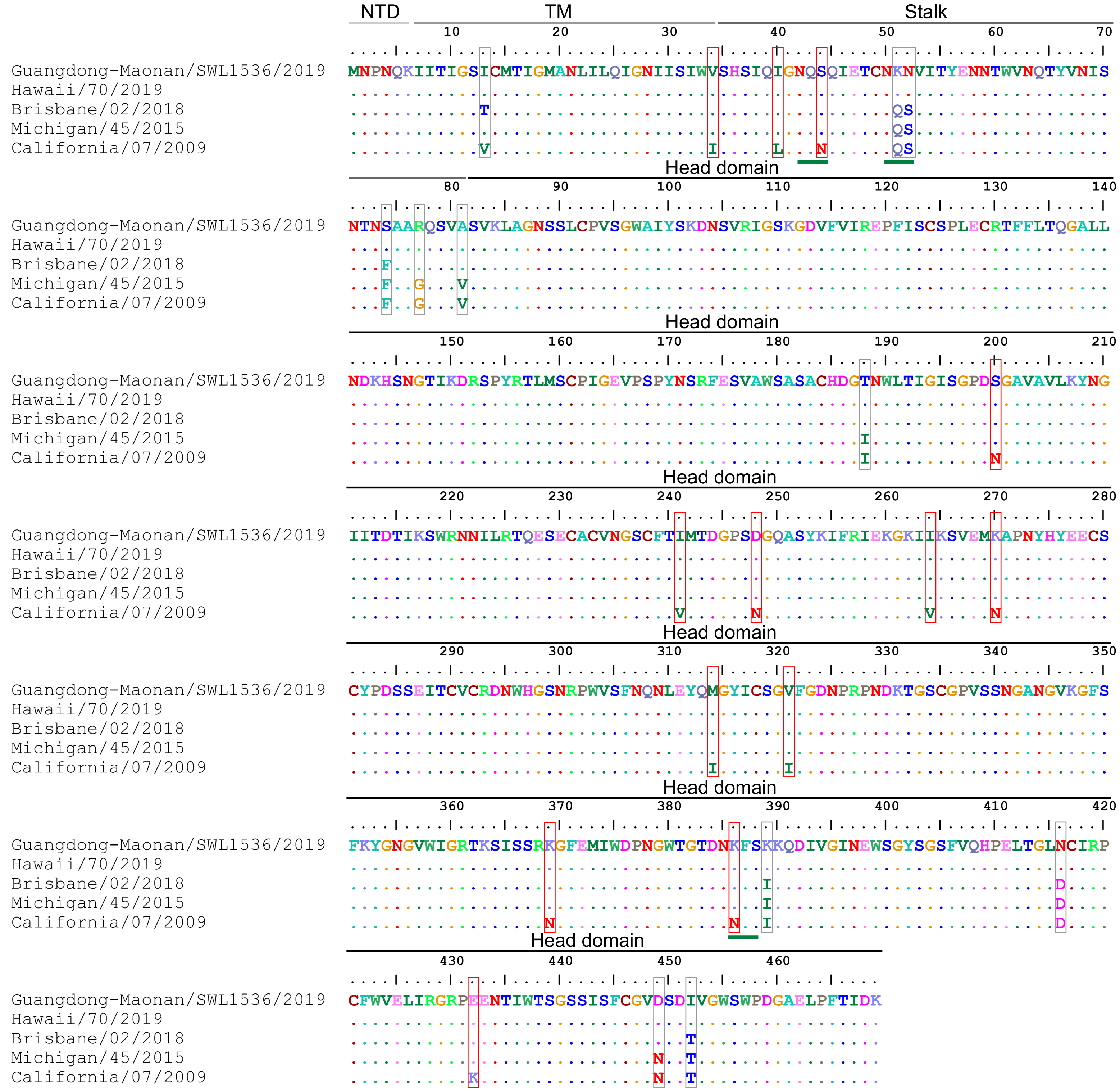

Supplementary Figure 1 | Alignment of NA sequences from recently recommended H1N1 vaccine strains. NA amino acid sequences from the indicated H1N1 IAV vaccine strains are shown. The strains were recommended for the northern hemisphere influenza vaccines for the 2010-2017 seasons (California/07/2009), 2017-2019 seasons (Michigan/45/2015), 2019-2020 season (Brisbane/02/2018) and for the 2020-2021 season (egg-based, Guangdong-Maonan/SWL1536/2019, and cell-based, Hawaii/70/2019, vaccines). Lines above the amino acid numbers indicate the residues comprising the short N-terminal domain (NTD), transmembrane domain (TM), stalk, and the enzymatic head domain. Red boxes highlight residues unique to the NA (N1-CA09) from the California/07/2009 vaccine strain, grey boxes indicate positions where two or more NAs differed from the NA (N1-GD19) in the Guangdong-Maonan/SWL1536/2019 vaccine strain. Green lines highlight substitutions in *N*-linked glycosylation sites (N-X-S or N-X-T) that varied between strains.

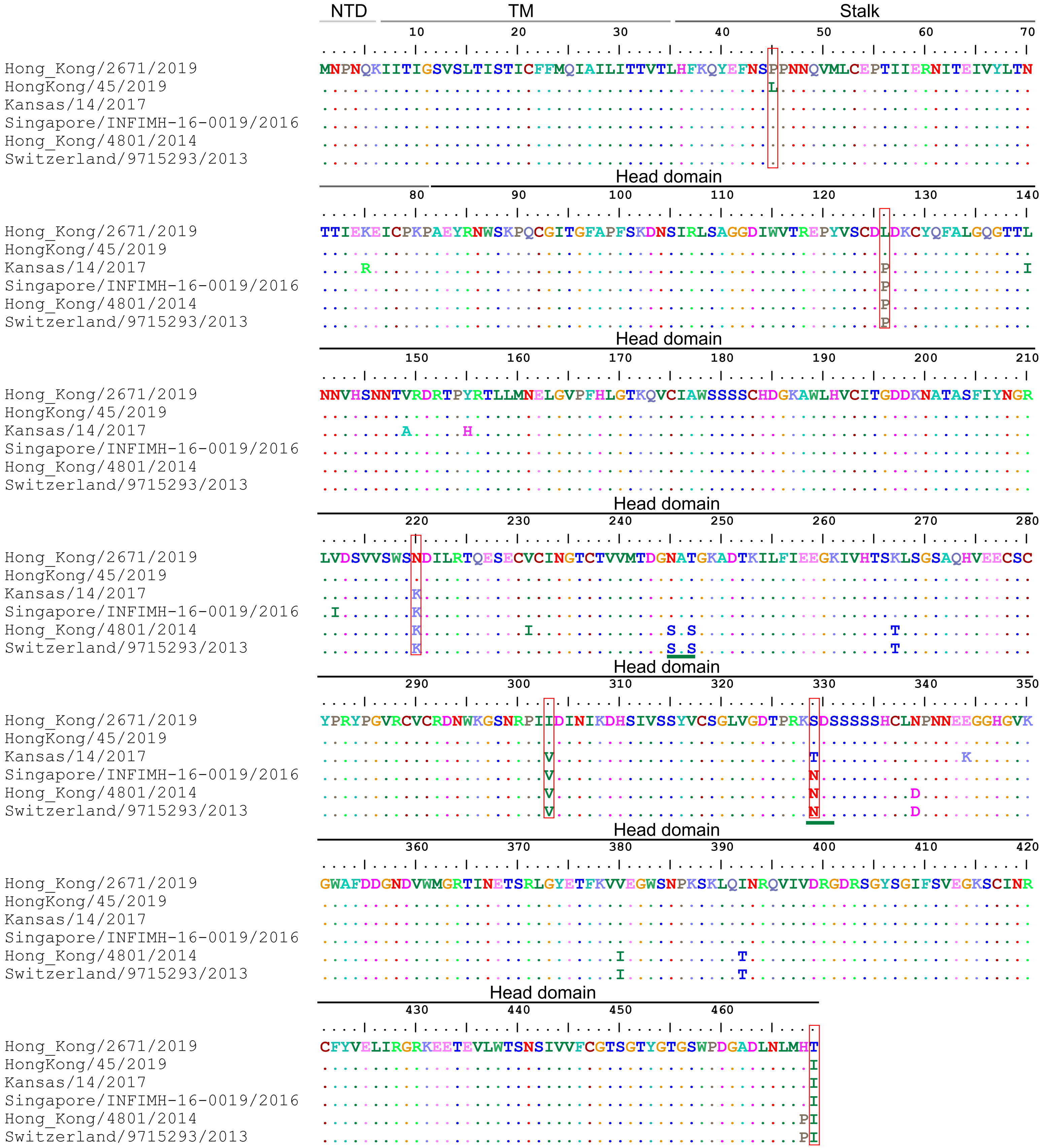

Supplementary Figure 2 | Alignment of NA sequences from recently recommended H3N2 vaccine strains. NA amino acid sequences from the indicated H3N2 vaccine strains are shown. The strains were recommended for the northern hemisphere influenza vaccines for the 2015-2016 season (Switzerland/9715293/2013), 2016-2018 seasons (Hong Kong/4801/2014), 2018-2019 season (Singapore/INFIMH-16-0019/2016), 2019-2020 season (Kansas/14/2017) and for the 2020-2021 season (egg-based, Hong Kong/2671/2019, and cell-based, Hong Kong/45/2019, vaccines). Lines above the amino acid numbers indicate the residues comprising the N-terminal domain (NTD), transmembrane domain (TM), stalk, and the enzymatic head domain. Red boxes highlight residues that are unique to at least one of the two NAs in the vaccine strains recommended for the 2020-2021 season. Green lines highlight substitutions in *N*-linked glycosylation sites (N-X-S or N-X-T) that varied bewteen strains.

a

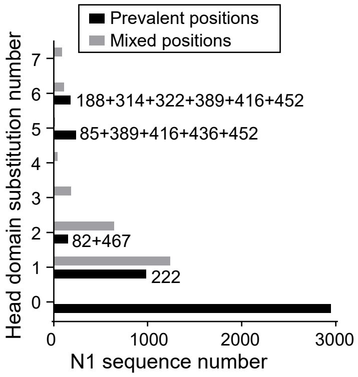

b

NA substitution compared to N1-GD19

H1N1 Strain	Head	Stalk	TM
Ontario/RV4818/2020	222K	52S* + 66F	19T
Arizona/12100/2020	82P + 467V	52S*	-
North Carolina/11896/2020	85I + 389I + 416D + 436V + 452T	51Q + 52S* + 53I + 74L	-
New Jersey/11588/2020	188I + 314I + 322L + 389I + 416D + 452T	51Q + 52S* + 74F	-

Supplementary Figure 3 | Sequence based selection of prevalent NAs from recent H1N1 IAVs. a, N1 sequences from recent human H1N1 IAVs (isolated between 09-01-2019 and 12-15-2020) were grouped based on the number of head domain substitutions with respect to N1-GD19. N1 sequences from each group that possessed the indicated amino acid substitution sites in the head domain were extracted and tabulated (black) together with the number of sequences that contain substitutions at different positions (grey). **b,** Chart containing the representative N1s selected for the antigenic comparison to N1-GD19 based on the position and prevalence of the head domain substitutions. Amino acid substitutions with respect to N1-GD19 are listed by the domain in NA. TM - transmembrane domain; *Substitution introduces a potential *N*-linked glycosylation site.

a

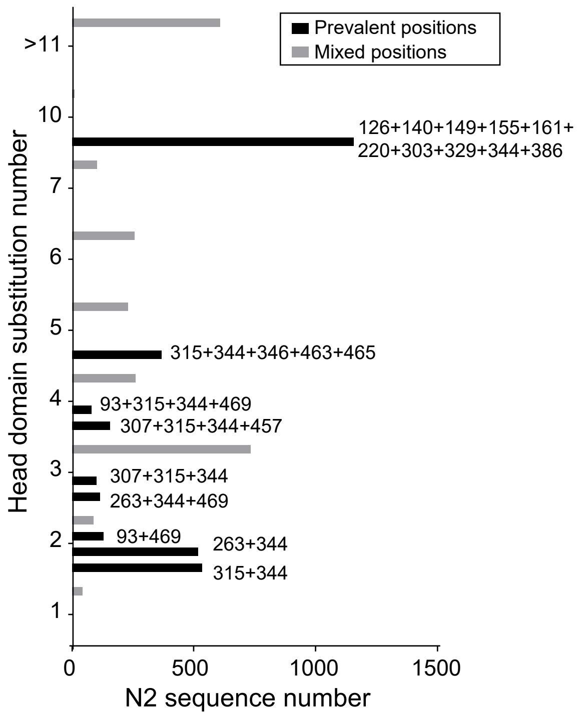

b

	NA substitution compared to N2-HK19c		
H3N2 Strain	Head	Stalk	TM
Virginia/12037/2020	93D + 469T[#]	45P[#]	-
Minnesota/24/2020	263I + 344K	45P[#]	-
Michigan/157/2020	315R + 344K	45P[#]	-
Pennsylvania/03/2020	263I + 344K + 469T[#]	45P[#]	-
California/33/2020	307L + 315R + 344K	45P[#]	-
Ohio/12141/2020	93D + 315R + 344K + 469T[#]	45P[#]	-
Massachusetts/07/2020	315R + 344K + 346D + 463N* + 465S*	45P[#]	-
*Quebec/RV3597/2020***	126P + 140I + 149A + 155H + 161S + 220K + 303V + 329T + 344K + 386S	45P[#] + 57M + 75R	-

Supplementary Figure 4 | Sequence based selection of NAs from recent human H3N2 IAVs. a, N2 sequences from recent human H3N2 IAVs (isolated between 09-01-2019 and 12-15-2020) were grouped based on the number of head domain substitutions with respect to N2-HK19c. N2 sequences from each group with the indicated amino acid substitution sites in the head domain (black) were extracted and tabulated together with the number of sequences that possessed substitutions at other sites (grey). **b,** Chart containing the representative N2s selected for antigenic comparison to N2-HK19c and N2-HK19e based on the position and prevalence of the head domain substitutions. Amino acid substitutions with respect to N2-HK19c are listed by the domain in NA. TM - transmembrane domain; [#]Substituiton not present in N2-HK19e; *Substituiton introduces a potential *N*-linked glycosylation site; **Sequence is more similar to N2-KS17 than N2-HK19c.

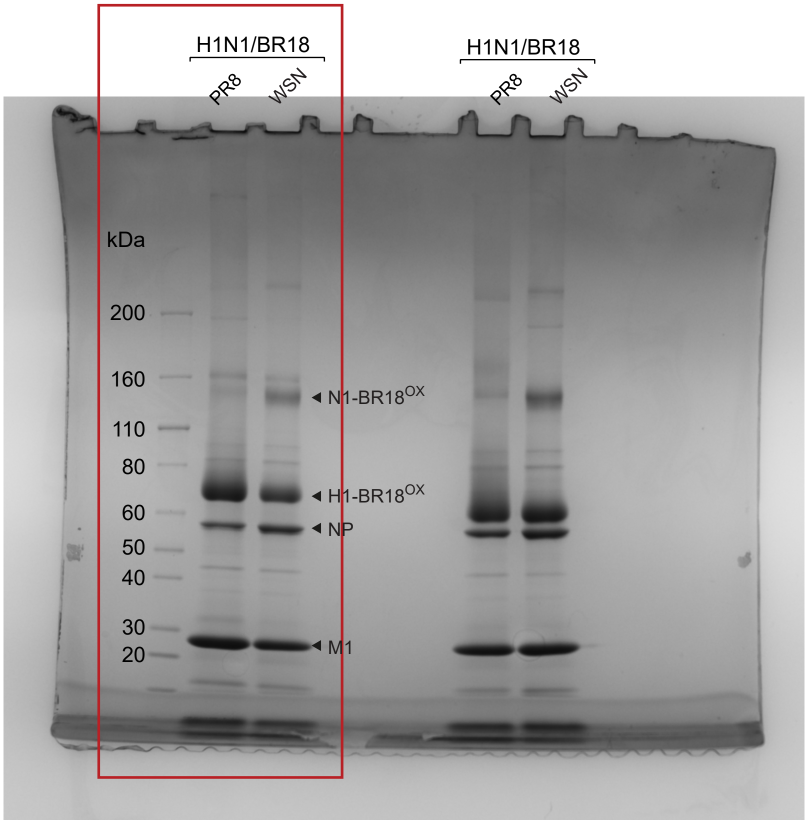

Supplementary Figure 5 | Unprocessed image of SDS-PAGE gel shown in Figure 2b. Original image of the Coomassie stained non-reducing SDS-PAGE gel (4-12%) shown in Fig. 2b is displayed. Approximately 5 µg of total protein for each of the indicated reassortant viruses was resolved. Lanes shown in Fig. 2b are indicated by a red box. The molecular weight marker is included for reference.
